# Reproducibility of [18F]FDG PET/CT liver SUV as reference or normalisation factor

**DOI:** 10.1007/s00259-022-05977-5

**Published:** 2022-09-27

**Authors:** Gerben J. C. Zwezerijnen, Jakoba J. Eertink, Maria C. Ferrández, Sanne E. Wiegers, Coreline N. Burggraaff, Pieternella J. Lugtenburg, Martijn W. Heymans, Henrica C. W. de Vet, Josée M. Zijlstra, Ronald Boellaard

**Affiliations:** 1 Radiology and Nuclear Medicine, Amsterdam UMC Location Vrije Universiteit Amsterdam, Amsterdam, The Netherlands; 2 Cancer Center Amsterdam, Imaging and Biomarkers, Amsterdam, The Netherlands; 3 Amsterdam UMC Location Vrije Universiteit Amsterdam, Hematology, Amsterdam, The Netherlands; 4 Erasmus MC Cancer Institute, University Medical Center, Hematology, Rotterdam, The Netherlands; 5 Epidemiology and Data Science, Amsterdam UMC Location Vrije Universiteit Amsterdam, De Boelelaan 1117, Amsterdam, The Netherlands; 6 Amsterdam Public Health Research Institute, Methodology, Amsterdam, The Netherlands

**Keywords:** Liver uptake, Reference, VOI, [18F]FDG PET/CT

## Abstract

**Introduction:**

Although visual and quantitative assessments of [18F]FDG PET/CT studies typically rely on liver uptake value as a reference or normalisation factor, consensus or consistency in measuring [18F]FDG uptake is lacking. Therefore, we evaluate the variation of several liver standardised uptake value (SUV) measurements in lymphoma [18F]FDG PET/CT studies using different uptake metrics.

**Methods:**

PET/CT scans from 34 lymphoma patients were used to calculate SUVmax^liver^, SUVpeak^liver^ and SUVmean^liver^ as a function of (1) volume-of-interest (VOI) size, (2) location, (3) imaging time point and (4) as a function of total metabolic tumour volume (MTV). The impact of reconstruction protocol on liver uptake is studied on 15 baseline lymphoma patient scans. The effect of noise on liver SUV was assessed using full and 25% count images of 15 lymphoma scans.

**Results:**

Generally, SUVmax^liver^ and SUVpeak^liver^ were 38% and 16% higher compared to SUVmean^liver^. SUVmax^liver^ and SUVpeak^liver^ increased up to 31% and 15% with VOI size while SUVmean^liver^ remained unchanged with the lowest variability for the largest VOI size. Liver uptake metrics were not affected by VOI location. Compared to baseline, liver uptake metrics were 15–18% and 9–18% higher at interim and EoT PET, respectively. SUV^liver^ decreased with larger total MTVs. SUVmax^liver^ and SUVpeak^liver^ were affected by reconstruction protocol up to 62%. SUVmax and SUVpeak moved 22% and 11% upward between full and 25% count images.

**Conclusion:**

SUVmean^liver^ was most robust against VOI size, location, reconstruction protocol and image noise level, and is thus the most reproducible metric for liver uptake. The commonly recommended 3 cm diameter spherical VOI-based SUVmean^liver^ values were only slightly more variable than those seen with larger VOI sizes and are sufficient for SUVmean^liver^ measurements in future studies.

**Trial registration:**

EudraCT: 2006–005,174-42, 01–08-2008.

**Supplementary Information:**

The online version contains supplementary material available at 10.1007/s00259-022-05977-5.

## Introduction

^18^F-fluoro-deoxy-glucose ([18F]FDG) positron emission tomography-computed tomography (PET/CT) is widely used for diagnosis, staging, response prediction, and monitoring in oncology and lymphoma. In the majority of cases, clinical reads are based on visual assessment of [18F]FDG uptake and distribution in lesions and across the body [1]. Yet, quantitative [18F]FDG PET assessments have gained interest, with quantitative uptake measures such as standardised uptake values (SUV), metabolic tumour volume (MTV), or total lesion glycolysis (TLG) showing diagnostic, prognostic and predictive value for several oncological and haematological applications [2–4].

Both visual evaluation and quantitative assessments of [18F]FDG uptake require standardisation and harmonisation of the [18F]FDG PET/CT examinations in order to obtain reproducible results [5]. To this end, various scientific organisations have issued [18F]FDG PET/CT imaging procedural guidelines and set up PET/CT accreditation or validation programs to assure that PET-CT systems are calibrated correctly and that certain reconstruction protocols are applied to guarantee harmonised image qualities and quantitation [6–8]. Despite these efforts, [18F]FDG PET/CT may still suffer from several uncertainties. Consequently, the European Association of Nuclear Medicine (EANM) guideline recommends to assess liver SUV for quality control (QC) purposes of the patient’s examination [6]. Mean liver SUV, derived from a 3-cm-diameter volume of interest (VOI) placed in the upper right lobe of the liver, as recommended by the EANM guideline, is expected to be within a range between SUV 1.3 and 3.0 [6]. A mean liver SUV outside this range may suggest errors in patient weight, injected activity or deviation in the performance of the PET/CT system. In case of visual reads, liver uptake is sometimes used as reference. For example, in lymphoma interim and end-of-treatment (EoT) PET studies, the so-called Deauville score is based on visually assessing whether and to what extent tumour uptake exceeds liver uptake (or mediastinal blood pool) [1, 9]. This assessment is used to classify tumour uptake into a 5-point score, which is subsequently used in clinical decision-making. Moreover, tumour contouring thresholds may be based on liver SUV, e.g. by taking the mean liver SUV times a factor as the SUV threshold to be used for lesion segmentation [10]. A similar approach is used by PERCIST to differentiate between target and non-target lesion for assessing treatment response using the mean liver SUV plus 2 standard deviations [11]. Thus, liver uptake is an important factor used for several purposes for both visual and quantitative [18F]FDG PET/CT reads.

There is, however, no consensus nor consistency in the way liver SUV is used. For QC purposes, the EANM, PERCIST, UPICT and SNMMI recommend to evaluate the mean SUV in a 3-cm-diameter VOI placed in the upper right lobe of the (unaffected) liver. The ACRIN trial protocols recommend the use of liver SUVmean calculated from a maximum region of interest (ROI) diameter in the liver [12]. In the approach for quantitative Deauville score at interim PET, is the lesion peak SUV (SUVpeak) compared with liver SUVmean to derive the quantitative Deauville score [13]. Interestingly, for EoT PET/CT in lymphoma patients, lesion maximal SUV (SUVmax) is compared with liver SUVmax [1, 14]. Likewise, there is also lack of a consistent definition of SUVpeak, which is derived by using a 1-mL spherical VOI as per EANM, PERCIST, SNMMI and QIBA recommendations versus the use of a certain number of connected hottest voxels near the SUVmax [15]. Although one VOI and SUV metric definition may not necessarily be better than the other, they are different and thus might generate different uptake values. Supplementary Table 1 provides an overview of the various liver uptake assessment methodologies as will be discussed later in this paper.

The inconsistency in the definition of liver uptake and the use of various VOI definitions makes comparisons across studies, even before, during and after treatment within the same patient, difficult. Moreover, the use of SUVmax is under debate as it has been reported that it is prone to noise, showing increased upward bias with elevated noise levels, and is more sensitive to variations in image quality and reconstruction settings/protocols than SUVpeak and SUVmean [16, 17].

The aim of this paper is therefore to perform an evaluation on the variation of several liver SUV uptake measurements arising from using different uptake metrics, different VOIs, different noise levels, at different time points across treatment (baseline, interim and EoT) and at different tumour loads. The paper will conclude by providing some recommendations for the use of liver SUV that will improve the robustness and reproducibility of liver SUV in multicentre studies and for comparisons between studies.

## Methods

### Patient datasets

In this study, three study datasets were used. The first dataset (dataset 1) consisted of 34 randomly selected patients with diffuse large B cell lymphoma (DLBCL) for whom baseline, interim and EoT scans were available from the HOVON-84 study (EudraCT2006-005,174–42, NTR1014) (Supplementary Table 2) [18]. Sites were instructed to perform the PET/CT studies following the EANM recommendation including a 4-h fasting status for patients before tracer administration, a 60-min ﻿﻿﻿[18F]FDG uptake interval and use of EARL1 standards accredited PET/CT systems. More details of the HOVON-84 study are described previously [18]. The institutional review boards approved the study at all centers, and participants gave written informed consent before enrollment.

For the second dataset, we used *n* = 15 ﻿﻿﻿[18F]FDG PET/CT scans of lymphoma patients scanned at baseline in our institute using a Philips Ingenuity PET/CT system (Philips Healthcare, Cleveland, USA). The latter scans were collected consecutively from ongoing clinical investigations and the use of these (anonymised) data for technical scientific purposes was waived by the VU Medical Center ethics review board. The data was used previously and are described in Kaalep et al. [19]. This dataset is presently being used to explore the impact of different image reconstruction protocols on liver SUV. In short, PET scans were performed conform EANM recommendation using a bed scan duration of 2 min and an injected activity of 3 MBq/kg. Scans were reconstructed following protocols compliant with EARL-1 and 2 standards; both implemented with 4 × 4 × 4 mm voxels. In addition, a locally preferred high-resolution reconstruction was included using 2 × 2 × 2 mm voxels in combination with so-called point spread function (PSF) reconstructions [17]. As available on the PET/CT system, the vendor-provided reconstruction algorithm (called BLOB-OS-TF) was used to generate the reconstructed PET images.

Finally, a third dataset was generated to explore the impact of noise on liver SUV. For this dataset, we included *n* = 15 new ﻿﻿﻿[18F]FDG PET/CT studies of lymphoma patients scanned at baseline using the Philips Ingenuity PET/CT system (Philips Healthcare, Cleveland, USA). Data were taken from ongoing routine clinical investigations, and the use of the (anonymised) data for technical scientific purposes was waived by the VU Medical Center ethics review board. Two sets of reconstructions were made. First, we reconstructed the full count image data taking all the counts acquired during our standard 2 min per bed position acquisition protocol using a 3MBq/kg FDG activity prescription (scan duration = 120 s per bed). Next, for each bed position, we used only the counts collected during the first 30 s for each bed position to generate an image with 25% of the full count, resulting in images with about 2 times higher noise level (4 times less counts gives about 2 times higher percentage noise).

### Image analysis and liver uptake measurements

In the first (HOVON-84) dataset, the liver uptake in all images was analysed in several ways with respect to VOI size and location. Spherical VOIs of 1, 1.5, 2, 3 and 5 cm diameters were placed in the (unaffected) right upper lobe of the livers. In addition, spherical VOIs of 3 cm diameter were placed at different locations within the liver, as illustrated in Supplementary Fig. 1. Liver SUVmax, SUVpeak and SUVmean were derived and will be reported as function of VOI size and location. SUVpeak was derived according to EANM guidelines using a 1-mL VOI. Moreover, the 3-cm diameter VOI-based liver SUVs were also used to study the impact of imaging time point, i.e. baseline, interim and EoT PET. For the HOVON-84 baseline scans, the total MTV was derived using the fixed SUV4.0 threshold, which was recently found to be preferred and robust to assess total tumour burden at baseline in DLBCL [18F]FDG PET/CT studies [20]. The total MTVs obtained were then compared to the observed liver SUV metrics to study if there is an association between liver SUV and tumour load.

For the second and third datasets, we used the 3-cm-diameter VOI placed in the upper right lobe of the liver, as per EANM recommendations. SUVmax, SUVpeak and SUVmean were reported for each of the applied reconstruction protocols for dataset 2 and measured on the 120 s full and 30 s (25%) count data using dataset 3 and directly compared to demonstrate the impact of reconstruction protocol and noise on liver SUV.

### Statistical analysis

Data is described using median values, interquartile ranges (IQR) and is presented using Tukey’s boxplots or scatter plots including generalized additive models (GAM) based trend lines. Significance of differences (*p* ≤ 0.05) among data was based on paired *t* tests or Wilcoxon signed rank test, when appropriate. Associations (*p* ≤ 0.05) were analysed using Spearman’s rank-order correlation testing.

## Results

**Fig. 1:**
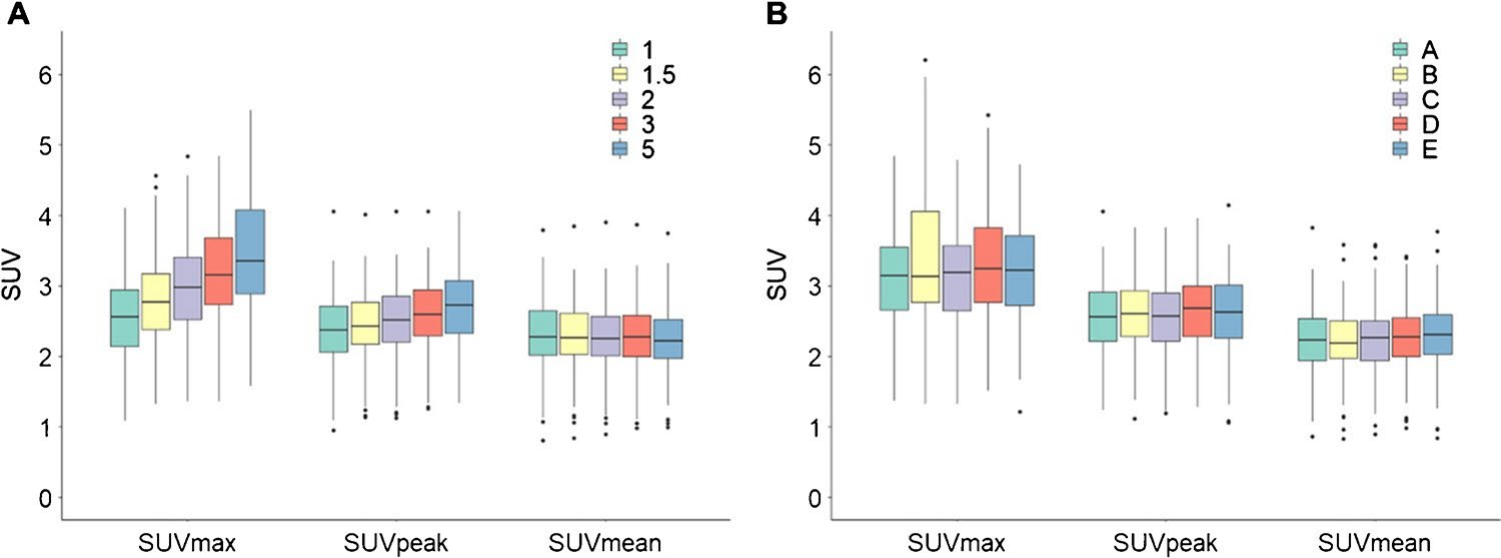
Liver SUV metrics as function of VOI size (**A**) and location (**B**): **A** SUVmax, SUVpeak and SUVmean per liver VOI size (cm). **B** SUVmax, SUVpeak and SUVpeak derived per VOI location in liver A-E (as visualized in Supplementary Fig. 1). Central line of the box is the median, edges of the box are the 25th and 75th percentiles, the whiskers extend to either of the most extreme data points, which are not considered outliers or 1.5 times interquartile range

Generally, liver SUVmax and SUVpeak were 38% and 16% higher in all analyses compared to SUVmean. The effects of VOI size and location are shown in Fig. 1a, b respectively. VOI size mostly affected SUVmax, which significant increased up to 31% as VOI size increased (1 vs 3 cm *p* < 0.001, 3 vs 5 cm *p* < 0.001), while SUVpeak only increased up to 15%, although significantly (1 vs 3 cm *p* < 0.001, 3 vs 5 cm *p* < 0.001) (Fig. 1a). SUVmean was the only metric that did not show a significant difference among the VOI sizes tested. Moreover, the liver SUVmax values were more variable with larger VOI sizes (IQR 0.80 at 1 cm increasing to IQR 1.19 at 5 cm). Contrarily, with increasing VOI sizes an overall decreasing trend in variability was seen when using SUVmean (IQR 0.67 at 1 cm and 0.50 at 5 cm). SUVpeak did not show a trend in variability with increasing VOI sizes (IQR 0.60 at 1.5 cm, IQR 0.65 at 1, 2 and 3 cm and IQR 0.74 at 5 cm). The VOI location (Supplementary Fig. 1) did not significantly affect the observed liver uptake measures (Fig. 1b). The SUVmax values were typically the highest (median ranges 2.6–3.3), followed by SUVpeak values (2.4–2.7) with SUVmean values generally being the lowest (2.2–2.3), regardless of VOI size or location (Supplementary Table 3).

**Fig. 2:**
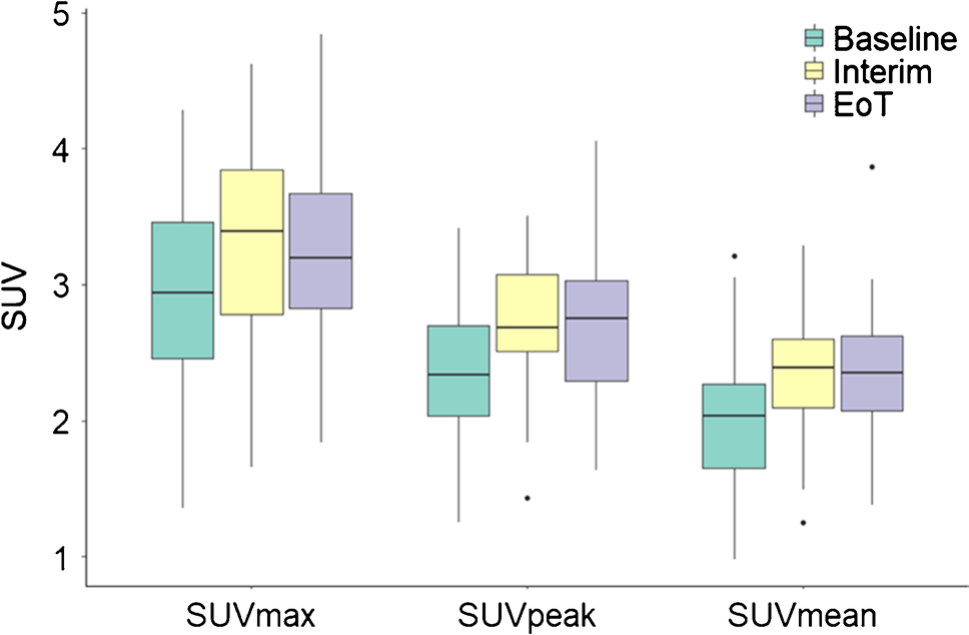
The effect of treatment time point (i.e. baseline, interim and end-of-treatment) on liver SUV metric values. Central line of the box is the median, edges of the box are the 25th and 75th percentiles, the whiskers extend to either of the most extreme data points, which are not considered outliers or 1.5 times interquartile range

As presented in Fig. 2, a consistent pattern of lower baseline SUV values for each liver uptake metric (SUVmax, SUVpeak and SUVmean) was observed when compared to interim and EoT PET/CT studies. Again, the SUV values differed between the metrics used with overall higher values for SUVmax (median ranges 2.9–3.4), followed by SUVpeak (2.3–2.7) and with the lowest values for SUVmean (2.0–2.4). Likewise, intersubject variability appeared to be largest when using SUVmax (IQR at baseline 1.0, interim 1.1, EoT 0.84) and generally smallest for SUVmean (IQR at baseline 0.62, interim 0.51, EoT 0.55).

**Fig. 3:**
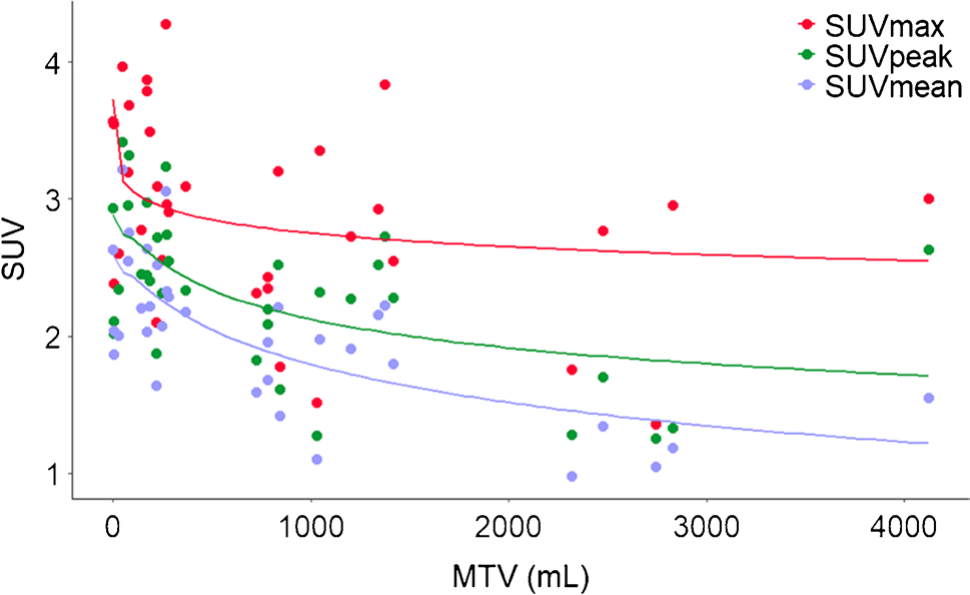
Liver SUV metric values as function of total metabolic tumour volume (MTV) in milliliter. Solid lines, which are data-driven flexibility curves implementing GAMs (generalised additive models), indicate the general trends

Liver SUV as function of total MTV is illustrated in Fig. 3. For all liver uptake metrics, a trend of decreasing SUV with increasing total MTV can be seen (all *p* < 0.05 using Spearman’s correlation testing) with again the largest intersubject variability when using SUVmax (IQR 1.0) versus SUVpeak (IQR 0.67) and SUVmean (IQR 0.62).

**Fig. 4:**
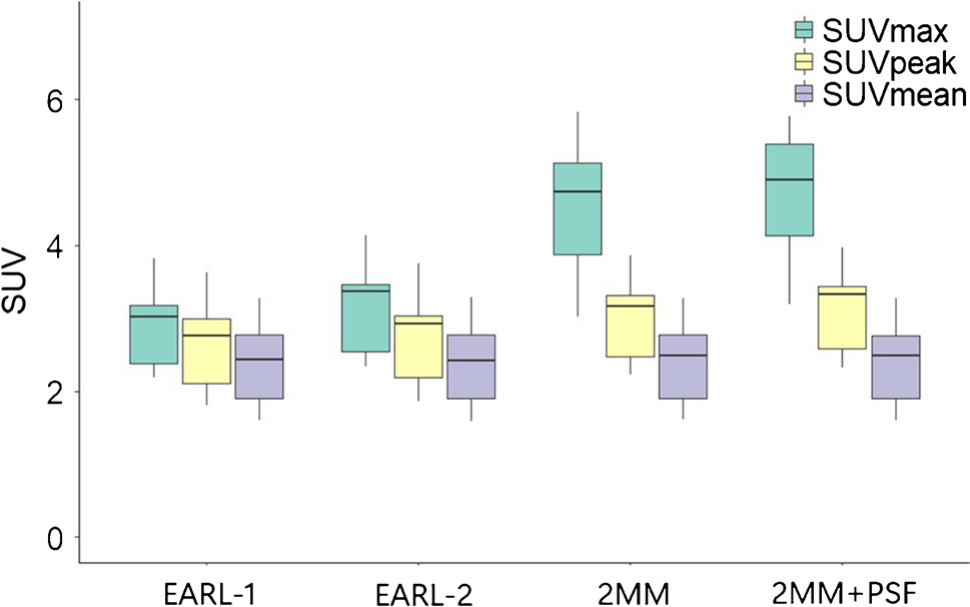
The effect of reconstruction protocol (EARL-1, EARL-2, 2 mm and 2 mm + PSF) on liver SUV metric values. Central line of the box is the median, edges of the box are the 25th and 75th percentiles, the whiskers extend to either of the most extreme data points, which are not considered outliers or 1.5 times interquartile range

**Fig. 5:**
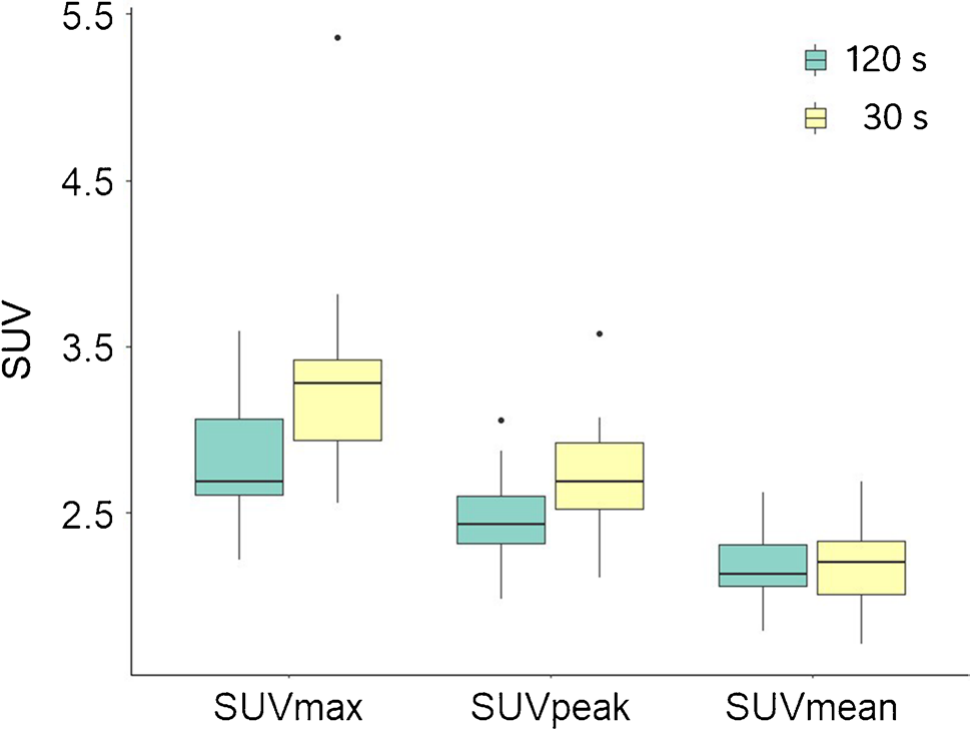
The effect of image noise (e.g. scan duration) on liver SUV metric values; full count images (120 s per bed position) versus 25% count images (30 s per bed position) images reconstructed on full count images. Central line of the box is the median, edges of the box are the 25th and 75th percentiles, the whiskers extend to either of the most extreme data points, which are not considered outliers or 1.5 times interquartile range

Finally, we studied the impact of reconstruction protocol (EARL-1, EARL-2, 2 mm, 2 mm + PSF) and the effect of image noise by means of scan duration on liver uptake. The liver SUVmax, and to a lesser extent the SUVpeak calculated on the EARL-1 reconstructed scans differed significantly compared to the liver uptake metrics using the other reconstructions (Fig. 4, Supplementary Table 3). The highest median liver SUVmax and SUVpeak values were observed when using 2 mm and 2 mm + PSF reconstructed scans (SUVmax 4.7 and 4.9, SUVpeak 3.2 and 3.3). In particular, the liver SUVmax values appeared to be most variable when derived on the latter reconstructions (IQR 1.26 and 1.27) compared to EARL-1 and EARL-2 (IQR 0.8 and 0.92). Reconstruction protocol barely affected the liver SUVmean values (median of 2.4–2.5). Figure 5 shows liver uptake SUVs measured on the 120 s (100%) and 30 s (25%) per bed position data. There was a clear and significant upward change in liver SUV between the 120 s and 30 s data, respectively, when using SUVmax (2.7 and 3.2, *p* < 0.05) and SUVpeak (2.4 and 2.7, *p* < 0.05), but not for SUVmean (2.1 and 2.2, *p* = 0.99).

## Discussion

In this study, we aimed to assess whether and to which extent VOI size and location, imaging time point related to treatment phase, tumour load, reconstruction parameters and image noise levels (e.g. scan duration) affect liver uptake measurements and to determine which image analyses approach—in terms of VOI definition and SUV metrics—would provide the most robust and reproducible assessment of liver SUV.

Our findings suggest that SUVmean based on a spherical VOI of 3 cm or larger seems to provide the most robust estimate of the underlying liver SUV, showing limited or no sensitivity for VOI size, VOI location and image noise (e.g. scan duration). Moreover, SUVmean for larger diameter VOIs showed least intersubject variability, most likely because averaging over a larger VOI volume reduces the impact of image noise. This can also be seen when using a 1-cm-diameter VOI (closely equal to the peak VOI definition), showing a similar level of variability as that of SUVpeak. SUVmax is based on a single voxel and therefore most sensitive to noise (increasing when applying PSF and/or smaller voxel size reconstruction and/or shorter acquisition time [17, 21]) resulting in both upward bias as well as increased variability, which was shown previously for tumour uptake measurements as well [16, 19]. The upward bias can be easily understood because with increasing VOI sizes, the probability of finding a noise induced higher maximum value increases, as was explained by Boellaard et al. [21–23].

The exact location of the VOI within the liver appears to have less of an effect on the measured liver SUVs. This is advantageous as usually the VOI is placed by an observer and likely the exact placement of the VOI suffers from intra- and interobserver variability. In our study, the different evaluated locations were well separated such that the translation in VOI placement is much larger than can be expected from any observer when applying clear definitions and rules for placing the VOI, i.e. in the upper right lobe of the unaffected liver. Clearly, locations with image artifacts near the diaphragm due to breathing motion, causing a spatial mismatch between PET and CT for attenuation correction, should be avoided as this will obviously result in inaccurate liver SUV measurements.

Unexpectedly, baseline liver SUVs appear to be lower than those observed at interim and EoT, regardless of the VOI and metric used. In an attempt to explain these findings, we explored the relation between liver SUV and tumour load by means of total MTV, with the hypothesis that large volume tumours (bulky and/or strongly disseminated) would act as a sink, reducing the availability of [18F]FDG to be taken off by the liver. Indeed, we observed that liver SUVs decrease with increasing total MTV suggesting that baseline tumour [18F]FDG uptake, which is intense and can have large MTVs, may result in lower liver SUV. However, when considering the actual SUVs, these baseline values align with the normal acceptable range suggested in the EANM guideline. Alternatively, it could be hypothesized that interim and EoT liver SUV is elevated due to treatment effects causing extra metabolic activity in the liver. A variety of biological factors potentially affects liver uptake measurements, which are summarized in Supplementary Table 4. The sample size and number of representatives for each potential biological factor in the currently used dataset 1 precludes meaningful analyses of these potential biological biases on liver SUV. The exact cause(s) of the change in SUV with the treatment time points remains unclear, but at least readers should be aware the liver SUV may not remain constant during or after treatment which may have consequences for its use as QC measure, to derive liver SUV-based contouring thresholds or when performing visual or semi-quantitative Deauville scoring.

Based on the findings in this paper we conclude and propose the following recommendations: (1) liver SUVmean is most robust to VOI size, reconstruction protocol and image noise. It therefore reflects the most reproducible liver uptake metric and is recommended when liver uptake is used for QC purposes, as reference for tumour assessment and tumour segmentation thresholding or when it is used as normalisation factor. (2) SUVmax shows an upward bias compared to the other SUV metrics in liver as well as in lesions, as was repeatedly shown in several publications [21, 22]. SUVmax should therefore not be used for uptake quantification in liver and lesions. (3) Lesional SUVpeak seems to be a good surrogate for SUVmax, while being less affected by noise and image reconstruction protocol as shown before [16, 17, 19]. SUVpeak is nowadays widely available in several image analysis tools and does not depend on the exact tumour contouring method, i.e. minimal or no observer variability as was the main reason to use SUVmax before instead of SUVmean. Thus, when using tumour to liver ratio’s, we recommend using tumour SUVpeak divided by the liver SUVmean based on a VOI size of at least 3 cm in diameter, in order to achieve the most robust and reproducible uptake metrics. (4) Finally, liver SUV is affected by total MTV and/or imaging time point and validity of recommended QC reference ranges may need to be reconsidered or, at least, kept in mind or verified for the specific trial/study at hand.

## Conclusions

In this paper, we evaluated the impact of several factors that can affect the liver uptake assessment. Liver SUVmax seemed to be most sensitive to VOI size, image noise and reconstruction protocol and should therefore not be used for quality control purposes, to define tumour segmentation thresholds or as reference or normalisation value, i.e. when assessing tumour to liver ratios. Liver SUVmean was most robust against these factors, showing smallest inter-subject variability as well. The commonly recommended liver SUVmean derived from a 3-cm-diameter spherical VOI was only slightly more variable than those seen with larger VOI sizes, which does not seem to justify an urgent adjustment of current guidelines. Finally, we observed that liver uptake may differ systematically between different time points across treatment, possibly caused by the high lesional uptake and large total MTV seen at baseline [18F]FDG PET/CT in DLBCL patients and/or treatment effect which directly affects liver metabolism. This phenomenon should be kept in mind when using liver SUV for any quantitative purpose of when scoring lesion uptakes.

### Supplementary Information

Below is the link to the electronic supplementary material.

Supplementary file1 (DOCX 64 KB)

Supplementary file2 (DOCX 43 KB)

Supplementary file3 (DOCX 24 KB)

Supplementary file4 (DOCX 28 KB)

Supplementary file5 (DOCX 61 KB)

## Data Availability

The datasets generated during and/or analysed during the current study are available from the corresponding author on reasonable request.
