# Supplementary figures and images for: Reproducibility of [18F]FDG PET/CT liver SUV as reference or normalisation factor
Source: Eur J Nucl Med Mol Imaging. 2022 Sep 27;50(2):486–93. doi: 10.1007/s00259-022-05977-5 (PMC9816285; doi:10.1007/s00259-022-05977-5)

**Supplementary Figure 1** Placing and naming the five VOIs (A-E) in the liver


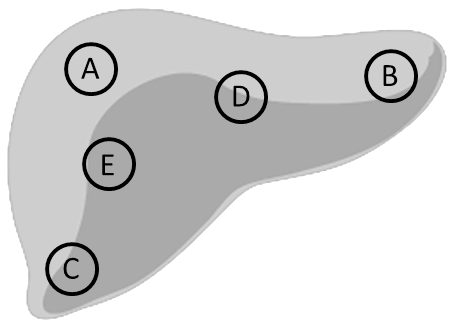

Supplement: Supplementary file 1 — Supplementary file1 (DOCX 64 KB) [file 259_2022_5977_MOESM1_ESM.docx]
