# Supplementary material for: Reproducibility of [18F]FDG PET/CT liver SUV as reference or normalisation factor
Source: Eur J Nucl Med Mol Imaging. 2022 Sep 27;50(2):486–93. doi: 10.1007/s00259-022-05977-5 (PMC9816285; doi:10.1007/s00259-022-05977-5)
Supplement: Supplementary file 2 — Supplementary file2 (DOCX 43 KB) [file 259_2022_5977_MOESM2_ESM.docx]

**Supplementary Table 1** Various liver uptake assessment methodologies

| Description | Application | VOI size or volume | SUVmetric | Location | Literature |
| --- | --- | --- | --- | --- | --- |
| PERCIST: SULpeak target lesion > 1.5 x SULmean liver + 2 SD | solid tumors | 3 cm spherical ROI | SULmean | right lobe | Wahl R.L. et al.[12] |
| qPET: SUVpeak target lesion > 1.5 x SUVmean liver + 2 SD | lymphoma | 30 cc cuboid (2:2:1:) | SUVmean | right lobe | Hasenclever D. et al.[24] |
| rPET: SUVmax target lesion > liver | lymphoma | 30 cc | SUVmax | right lobe | Annunziata S. et al.[25] |
| Peking criteria: SUVmax target lesion > liver | lymphoma | maximum ROI | SUVmax | not specified | Fan Y. et al.[26] |
| Deauville criteria: visual uptake and SUVmax target lesion > liver | lymphoma | not specified | SUVmax | not specified | Barrington S.F. et al.[2] |
| Quality Control; QIBA-UPICT, ACRIN, EANM/EARL | FDG PET QC | 3 cm diameter (ROI/VOI), max diameter ROI (ACRIN) | SUL/SUVmean | right upper lobe | Boellaard R. et al.[7], QIBA_UPICT_v113[9],  Scheuermann J.S. et al.[13] |
| Thresholding for tumor delineation and MTV assessment | solid tumor and lymphoma | 2-3 cm diameter | SUVmax, mean | right hepatic dome | Kanoun S. et al.[27], Vali F.S. et al.[28] , Kido H. et al.[5] Im H.J. et al.[29], Ilyas H. et al.[11], Hyun S.H. et al.[30] |
|  |  |  |  |  | Eude F et al.[31] |

QIBA-UPICT: Quantitative Imaging Biomarkers Alliance-Uniform Protocol for Imaging in Clinical Trials. ACRIN: American College of Radiology Imaging Network. EANM/EARL: European Association of Nuclear Medicine / EANM Research Ltd.
