# Supplementary material for: Reproducibility of [18F]FDG PET/CT liver SUV as reference or normalisation factor
Source: Eur J Nucl Med Mol Imaging. 2022 Sep 27;50(2):486–93. doi: 10.1007/s00259-022-05977-5 (PMC9816285; doi:10.1007/s00259-022-05977-5)
Supplement: Supplementary file 3 — Supplementary file3 (DOCX 24 KB) [file 259_2022_5977_MOESM3_ESM.docx]

**Supplementary Table 2** Patient characteristics of dataset 1. IQR: interquartile range.

|  | | **Dataset 1 (n=34)** |
| --- | --- | --- |
| Median age [years] (IQR) | | 64 (42-80) |
| Age > 60 years | | 23 (68%) |
| Males | | 18 (53%) |
| Median (IQR) blood glucose level [mmol/L] | Baseline | 5.6 (5.1-6.2) |
|  | Interim | 5.9 (5.6-6.6) |
|  | End-of-treatment | 5.6 (5.2-6.2) |
| Median (IQR) injection interval [min] | Baseline | 67 (60-79) |
|  | Interim | 63 (69-73) |
|  | End-of-treatment | 63 (60-71) |
| Median (IQR) BMI [kg/m²] | Baseline | 26.3 (24.6-28.3) |
|  | Interim | 25.7 (24.0-28.2) |
|  | End-of-treatment | 25.4 (24.1-28.0) |
| Median (IQR) Metabolic Tumor Volume [mL] | Baseline | 324 (169.8-1162.6) |
|  | Interim | 0.0 (0-0) |
|  | End-of-treatment | 0.0 (0-0.0) |
