# Supplementary material for: Reproducibility of [18F]FDG PET/CT liver SUV as reference or normalisation factor
Source: Eur J Nucl Med Mol Imaging. 2022 Sep 27;50(2):486–93. doi: 10.1007/s00259-022-05977-5 (PMC9816285; doi:10.1007/s00259-022-05977-5)
Supplement: Supplementary file 4 — Supplementary file4 (DOCX 28 KB) [file 259_2022_5977_MOESM4_ESM.docx]

**Supplementary Table 3** Significance of difference between liver SUV metrics per VOI size (1-5 cm), VOI location (A-E), image timepoint (baseline, interim and end-of-treatment (EoT), reconstruction protocol (EARL-1, EARL-2, 2MM, 2MM+PSF) and scan duration (120 s and 30 s per bed position).
