# Supplementary material for: Reproducibility of [18F]FDG PET/CT liver SUV as reference or normalisation factor
Source: Eur J Nucl Med Mol Imaging. 2022 Sep 27;50(2):486–93. doi: 10.1007/s00259-022-05977-5 (PMC9816285; doi:10.1007/s00259-022-05977-5)
Supplement: Supplementary file 5 — Supplementary file5 (DOCX 61 KB) [file 259_2022_5977_MOESM5_ESM.docx]

**Supplementary Table 4** Overview of potential biological factors influencing liver SUV measurements.

| **Biological factor potentially affecting liver uptake measures** | **FDG uptake** | **Literature** |
| --- | --- | --- |
| Male | decreased | Malladi, A. et al[32], Keramida, G., & Peters, A. M.[33] |
| Increased age | increased | Malladi, A. et al.[32], Lin, C. Y. et al.[34], Cao, Y. et al.[35] |
| BMI | increased | Mahmud, M. H. et al.[36], Malladi, A. et al.[32], Liu, G. et al.[37] |
| Increased basal glucose level | Increased | Wang, R. et al.[38], Sprinz, C. et al.[39], Malladi, A. et al.[32], Kubota, K. et al.[40], Keramida, G., & Peters, A. M.[41], Webb, R. L. et al.[42], Viglianti, B. L. et al.[43] |
| Low basal glucose level (≤120 mg/dl) | decreased | Rosica, D. et al.[44] |
| Insulin | decreased | Iozzo, P. et al.[45, 46] |
| Fasting | decreased | Tenley, N. et al.[47] |
| Bloodpressure | increased | Nam, H. Y. et al.[48] |
| Increased triglycerides | increased | Kamimura, K. et al.[49], Liu, G. et al.[50] |
| Decreased HDL cholesterol | increased | Kamimura, K et al.[49], Liu, G. et al.[50] |
| Fatty liver | increased | Liu, G. et al.[37], Keramida, G. et al.[51, 52], Alexander, D., et al.[53] |
| Hyperthyroidism | decreased | Chen, Y. K. et al.[54], Yang, P. et al.[55] |
| Liver function (increased AST, ALT, y-GT) ↓ | increased | Liu, G. et al.[50], Lin, C. Y. et al.[56], Keramida, G. et al.[51] |
| Increased tumor staging / higher MTV | decreased | Agostino, C., et al.[57], Oliveira, M. et al.[58], Kim, S. J. et al.[59], |
| Chemotherapy | increased | Wu, X. et al.[60], Ceriani, L. et al.[61], Chiaravalloti A et al.[57] |
| Steroid use | increased | Furuya, S. et al.[62] |
| Delayed uptake time | decreased | Alin, C., et al.[63], Mahmud, M. H. et al.[36], Malladi, A. et al.[32], Wang, R. et al.[64] |
